# Data-driven classification of playing styles and match outcome prediction in UEFA Champions League teams

**DOI:** 10.5114/biolsport.2026.154944

**Published:** 2025-11-03

**Authors:** Yonghan Zhong, Ying Xu, Kecheng Zhu, Jorge Diaz-Cidoncha Garcia, Miguel Ángel Gómez Ruano, Qing Yi

**Affiliations:** 1 College of Physical Education, Dalian University, 116622 Dalian, China; 2 School of Athletic Performance, Shanghai University of Sport, 200438 Shanghai, China; 3 Applied Technology College of Dalian Ocean University, 116023 Dalian, China; 4 Fédération Internationale de Football Association FIFA-Strasse 20 P.O. Box 8044 Zurich Switzerland; 5 Facultad de Ciencias de la Actividad Física y del Deporte-Inef Madrid, Universidad Politécnica de Madrid, Madrid, Spain

**Keywords:** Football, Playing style, Machine learning, Feature selection, Match analysis

## Abstract

This study proposes a data-driven framework for classifying UEFA Champions League teams into possession-based and counterattacking styles and predicting match outcomes based on key performance indicators (KPIs). Dimensionality reduction via an autoencoder was combined with K-means clustering to identify underlying tactical patterns beyond traditional possession metrics. Feature selection was performed using LASSO, Boruta, and XGBoost to determine the most relevant KPIs. Predictive models, including Support Vector Machine (SVM), K-Nearest Neighbors (KNN), and LightGBM, were evaluated using AUC and F1 Score. SVM achieved the highest performance for possession-based teams, whereas KNN outperformed other models for counterattacking teams. The results revealed distinct style-specific performance profiles. For possession-based teams, higher possession and key passes correlated negatively with winning probability, while crosses and long-range shots were positively associated with success. In counterattacking teams, increased possession and key passes improved match outcomes, whereas crosses and shots from outside the box showed negative associations. Defensive actions, particularly clearances, were strongly associated with improved defensive stability and match success, especially among counterattacking teams. This framework improves the accuracy of tactical classification and provides interpretable associations between KPIs and match outcomes. The findings can inform style-specific tactical planning and performance monitoring, enabling coaches to adjust offensive or defensive training priorities according to team strategy.

## INTRODUCTION

Performance in football matches is primarily determined by a combination of physical, technical, and tactical factors [1]. These factors can be assessed by analyzing how players attack and defend and by comparing the playing styles of the competing teams [2]. The increasing availability of big data in professional football now allows match performance to be examined systematically and in greater detail [3].

A key tactical factor in football is a team’s playing style [4], which refers to the typical behavioral patterns exhibited by players at the team level, that is, the repeated ways they play over time [4]. Playing styles are generally divided into offensive (in possession) and defensive (out of possession) phases, as player objectives and actions differ markedly in each [4]. The long-standing dichotomy between possession-play and direct-play, although widely used in performance analysis, has often lacked conceptual clarity. A recent grounded theory synthesis reviewed and integrated over 200 academic sources to produce a taxonomy of playing styles grounded in empirical literature. This framework validates the classification of team strategies across and within game phases and supports the distinction between possession-oriented and counterattacking approaches as a consistent and theoretically justified dichotomy [5]. Possession-play and direct-play require different tactics, skills, and physical attributes [6]. As a result, certain performance indicators frequently emerge as reliable measures of team strategy and overall style of play [7].

Teams characterized by possession-play typically control the ball for longer periods, complete more passes, and maintain higher possession rates than their opponents [8]. Direct-play teams, in contrast, rely on long passes, rapid transitions, and reduced possession time [4]. Possession rate is thus commonly used to differentiate tactical styles [9]. However, reliance on possession alone overlooks other crucial aspects of strategy, including defensive organization, transition efficiency, and shot conversion [7, 10]. Even possessionoriented teams depend on pressing intensity, passing dynamics, and shot selection to achieve success [9]. Therefore, possession rate provides only a basic classification and oversimplifies the complexity of playing styles by focusing solely on ball control. A more comprehensive understanding requires integrating additional performance indicators that capture tactical priorities and execution on the field [11].

Machine learning (ML) has advanced rapidly in both applications and methodologies [12]. Unlike traditional statistical approaches, ML algorithms impose fewer assumptions on the data and are more effective for modeling complex patterns [13]. Playing style analysis has commonly employed multivariate techniques such as factor analysis, principal component analysis (PCA), and k-means clustering [14]. However, PCA is a linear method and often fails to capture nonlinear relationships, limiting its effectiveness in identifying playing styles [15]. Neural network-based methods, such as autoencoders, can better capture these nonlinear patterns [16].

Despite advances in data availability, a significant methodological gap remains: the absence of an integrated, nonlinear, and explainable machine learning framework capable of simultaneously classifying playing styles and predicting match outcomes. Most prior studies treated tactical classification and result prediction as separate tasks. This study addresses the need for a unified approach that captures the multidimensional, nonlinear, and interactive nature of football performance.

Football involves dynamic interactions across micro-, meso-, and macro-levels that are poorly represented by traditional linear models [17]. To address this limitation, nonlinear dimensionality reduction using an autoencoder is employed to extract latent patterns from high-dimensional technical indicators. This approach detects subtle, nonlinear structures that linear reductions or predefined thresholds (e.g., possession-rate cutoffs) fail to capture. Unsupervised Kmeans clustering is then applied to the encoded features to identify emergent playing styles without relying on manually defined labels, thereby avoiding the oversimplification of tactical behaviors.

Previous approaches relied on style archetyping based on predefined variables (e.g., possession rate or passing frequency), often using linear assumptions or single-indicator thresholds. These methods overlook the multidimensional and nonlinear characteristics of tactical behavior. In contrast, this study combines autoencoder-based nonlinear dimensionality reduction with unsupervised clustering to derive playing styles directly from performance data. The objectives are to identify data-driven playing styles without predefined labels and examine how key performance indicators (KPIs) influence match outcomes under different tactical approaches. Specifically, the study explores whether performance metrics contribute differently to success in possession-based versus counterattacking teams.

The proposed framework integrates autoencoder-based feature extraction with K-means clustering for style classification. A robust feature selection process combining LASSO, Boruta, and XGBoost identifies key predictors of success. Match outcomes are then predicted using three supervised models: support vector machine (SVM), k-nearest neighbor (KNN), and LightGBM. This integrated approach improves both interpretability and predictive accuracy in tactical analysis of elite football.

## MATERIALS AND METHODS

Technical performance and match outcome data for UEFA Champions League teams from the 2017/2018 to 2023/2024 seasons were collected from WhoScored.com (http://www.whoscored.com), which sources its data from OPTA Sportsdata, known for high interoperator reliability (kappa > 0.90) [18]. A total of 869 matches (n = 1,738 observations) were included based on data availability from the official UEFA website. Variables with excessive zero values were excluded to ensure reliable analysis, resulting in 30 technical performance variables retained for statistical modeling. As no human or animal participants were involved, no informed consent or ethical approval was required, consistent with the Declaration of Helsinki.

### Feature engineering

#### Clustering

A combination of autoencoder-based feature extraction and K-means clustering was used to categorize matches based on technical performance. Z-score standardization was applied to normalize feature scales [19].

Direct application of K-means to high-dimensional data is prone to suboptimal performance due to noise, redundancy, and the curse of dimensionality. To address these issues, an autoencoder was trained to learn a compact, lower-dimensional representation of the input variables. Autoencoders effectively capture nonlinear relationships and complex patterns while reducing noise and redundancy [16]. The autoencoder used a symmetrical neural network architecture with two components: the encoder and the decoder [20]. The encoder compresses high-dimensional input into a lower-dimensional latent space, while the decoder reconstructs the original input from the latent representation.

The encoded features were subsequently clustered using K-means to group matches based on underlying technical performance patterns.

#### Feature selection

An initial set of 30 key performance indicators (KPIs) from WhoScored was considered, without assuming any direct relationship with match outcomes. Dimensionality reduction and feature selection were performed using LASSO, Boruta, and XGBoost to identify the most informative predictors [21]. Only these selected variables were used in the predictive modeling framework.

Match outcomes were treated as a binary variable: WIN (3 points) and NOWIN (draw or loss, 0 points). Although a three-class classification (win, draw, loss) provides greater detail, previous studies have shown that draws are difficult to predict due to their stochastic nature and low frequency [22, 23, 24]. This introduces class imbalance and reduces model stability [25]. Binary classification offers more robust and interpretable models, particularly with high-dimensional performance data. Therefore, match outcomes were recoded into two categories: WIN and NOWIN (comprising both draws and losses). The dichotomous outcome variable aligns with the study’s primary objective, that is, to identify the KPIs most strongly associated with achieving victory.

Technical performance variables were used as predictors, while match outcomes served as the dependent variable. LASSO regression was applied to select relevant features and reduce model complexity by introducing a regularization parameter (λ), which penalizes less important coefficients and prevents overfitting. The optimal λ value (λmin) was determined through 10-fold cross-validation, with the minimum prediction error guiding the selection of key variables [26]. In contrast with conventional algorithms, Boruta, a random forest-based feature selection method, identifies all relevant predictors by comparing their importance against randomly generated “shadow” attributes. It iteratively evaluates the contribution of each feature and retains only those with a statistically significant impact on prediction [27, 28]. Extreme Gradient Boosting (XGBoost), an advanced ensemble learning method based on decision trees, was also employed. By combining multiple weak classifiers through iterative learning, XGBoost constructs a highly accurate model that captures nonlinear relationships and complex interactions among variables, making it particularly effective for classification tasks [29].

To determine the most robust key performance indicators (KPIs) for each tactical group, the clustering results were combined with the three feature selection techniques. LASSO prioritized model sparsity by removing irrelevant variables, Boruta identified nonlinear and hidden associations based on random forest principles, and XGBoost detected intricate variable interactions. The intersection of features selected by all three methods was used to ensure reliability and interpretability, striking a balance between parsimony and coverage. This integrative strategy retained only those KPIs consistently recognized across the different algorithms [30].

### Statistical analysis

Following feature selection, three ML models, namely Support Vector Machine (SVM), k-Nearest Neighbors (KNN), and LightGBM, were developed using the identified key features. These models were selected based on their distinct methodological characteristics and suitability for binary classification tasks. The dataset was randomly partitioned into training (70%) and testing (30%) subsets for model evaluation.

For the SVM model, a radial basis function (RBF) kernel was used. Hyperparameters (gamma and cost) were optimized via grid search combined with cross-validation, and the model yielding the best cross-validation performance was selected. For the KNN model, values of k ranging from 1 to 100 were tested using 10-fold cross-validation, with the value achieving the highest area under the receiver operating characteristic curve (AUC) selected. For the LightGBM model, gradient boosting was implemented with the following key hyperparameters: a learning rate of 0.02, 31 leaves (num_leaves), and early stopping after 30 iterations.

The models were evaluated and compared using two performance metrics: AUC and F1 score. The optimal model was selected based on these criteria, and its performance was further validated to confirm reliability and robustness [31].

To interpret the predictions of the selected model, SHapley Additive exPlanations (SHAP) values were computed to quantify the contribution of each feature. SHAP values offer a transparent and consistent measure of feature importance by explaining the direction and magnitude of each feature’s effect on the model’s output. This enhances the interpretability of the results and supports the relevance of the selected features in predicting match outcomes [32].

An ablation study was performed using a fixed random seed and a 70/30 train–test split to investigate the contributions of feature selection. Three classifiers—SVM, KNN, and LightGBM—were evaluated. For each classifier, two ablation settings were compared: E0 (Without feature selection), which used all raw features without any selection, and E1 (Full method), which combined autoencoderbased dimensionality reduction with multi-method feature selection. Model performance was assessed on the held-out test set using AUC and F1 scores.

All statistical analyses were conducted using R (version 4.4.3) and Python (version 3.10.9).

## RESULTS

### Analysis of the clustering results on reduced data

The quality of clustering and the optimal number of clusters (K) were evaluated using the silhouette score, which ranges from −1 to 1 and measures how well each data point fits within its assigned cluster compared to other clusters [33]. A higher score indicates betterdefined clusters. Silhouette scores were computed for different K values, and the number of clusters corresponding to the highest score was selected as optimal [33].

**FIG. 1:**
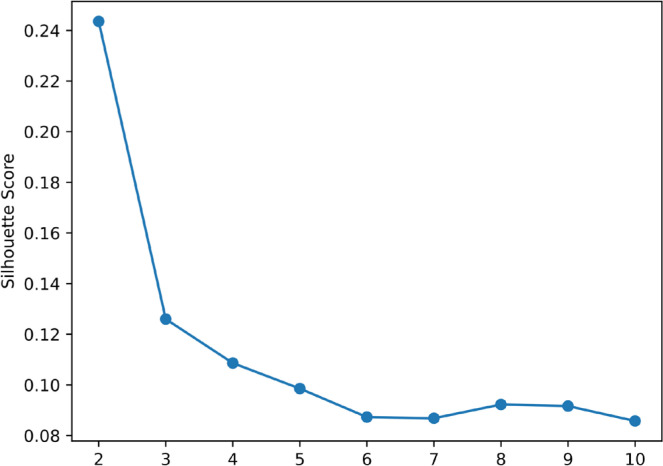
Silhouette score for optimal K.

The data were standardized before training an autoencoder to perform dimensionality reduction. After multiple training iterations, the encoder output stabilized, producing 2D feature representations. These low-dimensional representations were then used for clustering. Figure 1 shows the relationship between K and the corresponding silhouette scores obtained using the K-means algorithm.

The silhouette score was highest at K = 2, indicating that two clusters provided the best separation.

The two team styles identified through clustering were labeled Cluster 0 and Cluster 1. Descriptive statistics for key performance indicators (KPIs) in each cluster are presented in Table 1. Cluster 0 exhibited higher offensive metrics, including possession (mean = 55.6), key passes (mean = 10.9), and open-play shots (mean = 10.3), indicating a possession-based style. Cluster 1 displayed stronger defensive performance, with higher successful tackles (mean = 17.8), clearances (mean = 22.4), and defensive aerial duels (mean = 13.5), suggesting a counterattacking approach. This classification effectively differentiates possession-oriented teams from counterattacking teams.

**TABLE 1:** Descriptive statistics of performance indicators by team style.

Variable	0 (n = 1106)	1 (n = 632)
Possession (%)	55.6	40.1
Touches	750.2	570.5
Pass Success (%)	85.7	76.5
Accurate Passes	486.5	291.7
Key Passes	10.9	7.9
Total Passes	588.4	397.4
Crosses	17.7	13.2
Short passes	517.5	323.7
Dribbles Won	10.8	8.6
Dribbles Attempted	18.9	16.4
Dribble Success (%)	57.2	52.5
Dispossessed	9.6	9.1
Open Play	10.3	7.3
Set Piece	3.2	2.7
18-yard Box (%)	58	52.5
Aerials Won (%)	52.1	46.4
Successful Aerials	12.2	13.5
Defensive Aerials	11.9	13.5
Offensive Aerials	11.1	15.6
Successful Tackles	16.2	17.8
Tackles Attempted	25.6	28.7
Clearances	14	22.4
Interceptions	10.5	12.2
Was dribbled	9.5	11
Cards per Foul (%)	15.6	21.5
Out of Box (%)	35.3	41.4
Long Balls	51.4	59.4
Tackle Success (%)	63.8	62.5
Corners Accuracy (%)	45.5	47.6
Fouls	11.8	11.8

#### KPIs for different playing styles

**FIG. 2:**
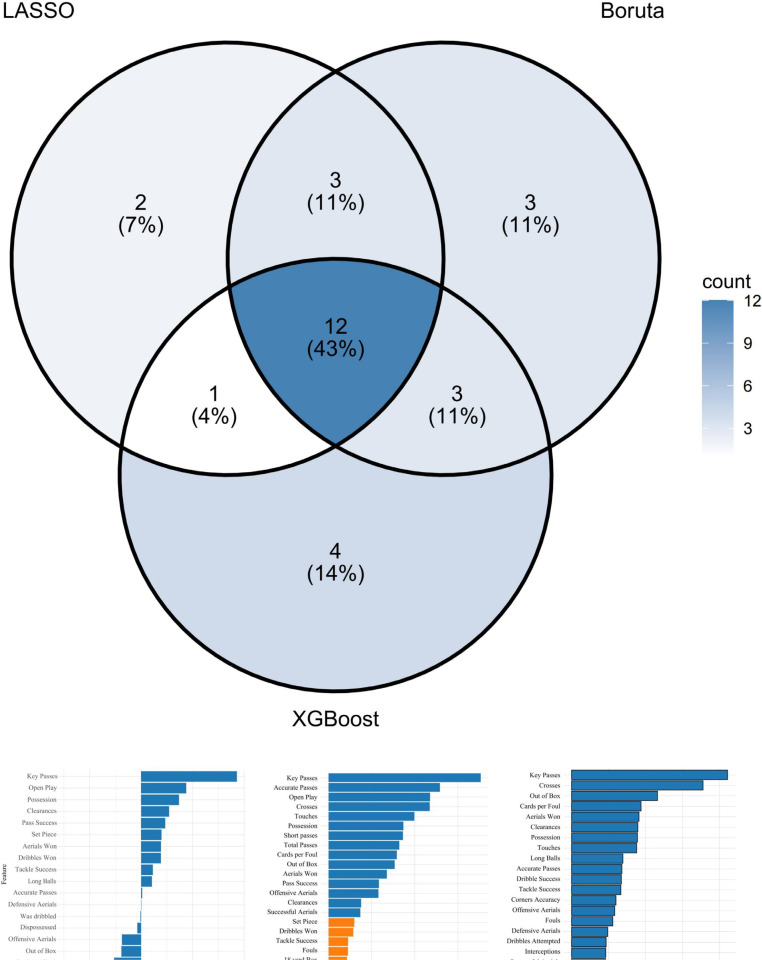
Feature importance and intersection analysis for possession-based style.

Feature selection was performed separately for the two playing styles to identify the most important indicators (Table 2). For the possession-based style, the LASSO model with λ_min_ = 0.0113 identified 18 predictive variables with non-zero coefficients. The Boruta algorithm iteratively removed statistically irrelevant features and, after 100 iterations, retained 21 relevant variables. XGBoost, with its faster computation and strong generalization, identified 20 predictive variables (Figure 2).

**TABLE 2:** Key indicators for different styles

Style	Indicator
Possession-based	Possession, Accurate Passes, Key Passes, Dribbles Won, Aerials Won, Offensive Aerials, Defensive Aerials, Tackle Success, Clearances, Out of Box, Crosses, Cards per Foul
Counterattacking	Possession, Key Passes, Defensive Aerials, Clearances, Open Play, Out of Box, Crosses, Cards per Foul

**FIG. 3:**
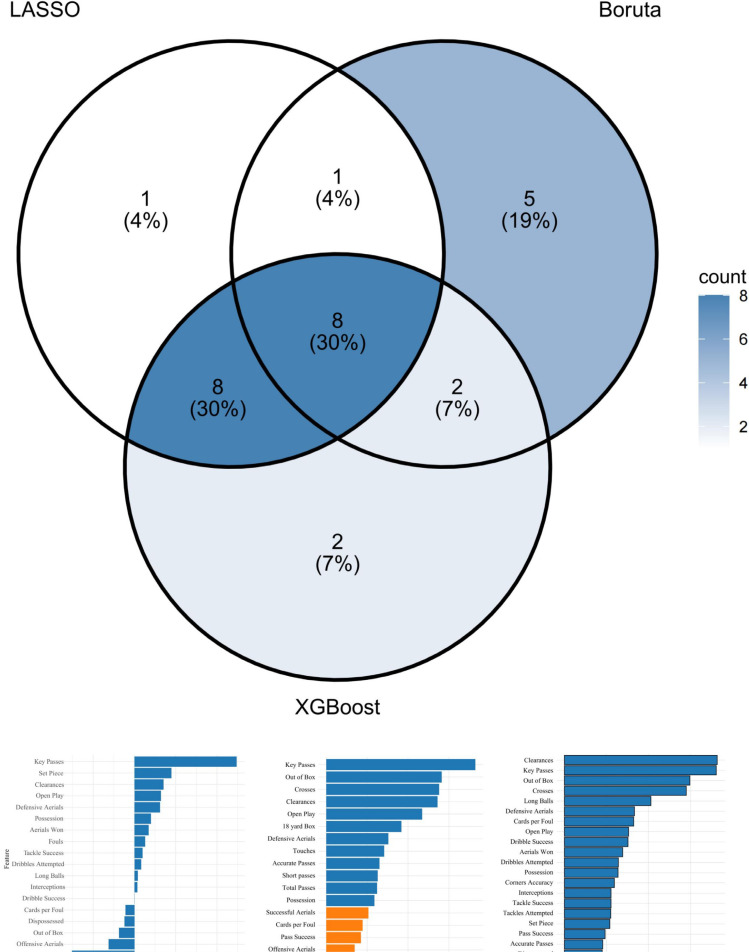
Feature importance and intersection analysis for counterattacking style.

For the counterattacking style, the LASSO model with λmin = 0.0095 selected 18 key indicators. Boruta retained 16 relevant features, while XGBoost identified 20 predictive variables (Figure 3).

### Model development and performance comparison

Table 3 compares the predictive performance of SVM, KNN, and LightGBM models using AUC and F1 scores for both possession-based and counterattacking scenarios, including ablation studies for each model. The results show that, for possession-based teams, SVM achieved the highest performance under E1 (Full method; AUC 0.783, F1 0.757), while performance under E0 (Without feature selection) was slightly lower (AUC 0.768, F1 0.743). KNN also performed better under E1 than E0 (AUC 0.735 vs 0.708; F1 0.718 vs 0.655). For counterattacking teams, KNN achieved the highest F1 under E1 (0.854) with an AUC of 0.726, whereas E0 resulted in lower performance (AUC 0.692, F1 0.645). LightGBM showed high sensitivity to feature selection, with AUC increasing from 0.717 to 0.741 but F1 decreasing from 0.683 to 0.235. Overall, these results indicate that the impact of feature selection varies across models and team types, with SVM performing best for possession-based teams and KNN for counterattacking teams.

**TABLE 3:** Model performance (AUC and F1 Score) including ablation study.

Model / Setting	Possession-based AUC (95% CI)	Possession-based F1 (95% CI)	Counterattacking AUC (95% CI)	Counterattacking F1 (95% CI)
SVM (E0)	0.768 (0.742–0.794)	0.743 (0.712–0.774)	0.746 (0.697–0.785)	0.732 (0.702–0.762)
SVM (E1)	0.783 (0.759–0.807)	0.757 (0.728–0.785)	0.701 (0.672–0.729)	0.840 (0.812–0.867)
KNN (E0)	0.708 (0.700–0.756)	0.655 (0.638–0.679)	0.692 (0.663–0.717)	0.645 (0.615–0.675)
KNN (E1)	0.735 (0.708–0.761)	0.718 (0.685–0.750)	0.726 (0.698–0.752)	0.854 (0.827–0.880)
LightGBM (E0)	0.759 (0.735–0.794)	0.693 (0.664–0.721)	0.717 (0.688–0.742)	0.683 (0.669–0.721)
LightGBM (E1)	0.774 (0.749–0.798)	0.671 (0.634–0.707)	0.741 (0.716–0.767)	0.235 (0.196–0.274)

Note: SVM - support vector machine; KNN - k-nearest neighbor; E0 - Without feature selection; E1 - full method.

**FIG. 4:**
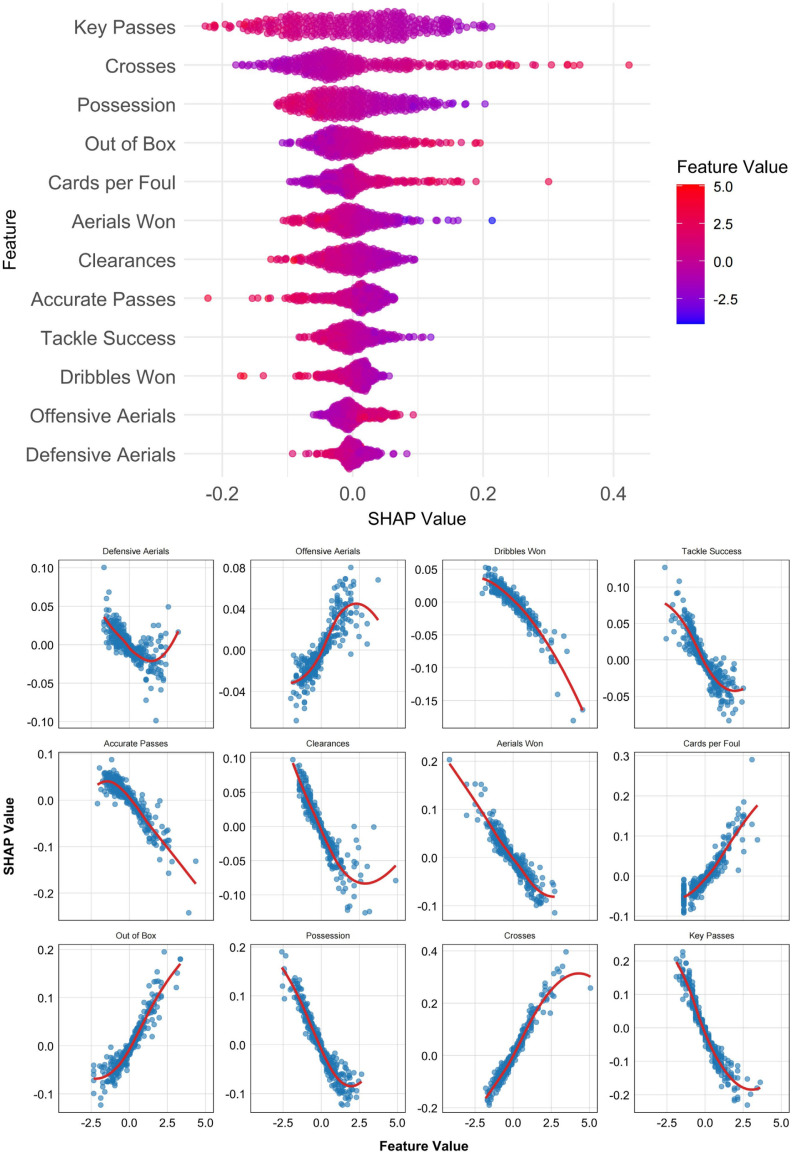
SHAP analysis of possession-based style: feature importance and dependence relationships.

**FIG. 5:**
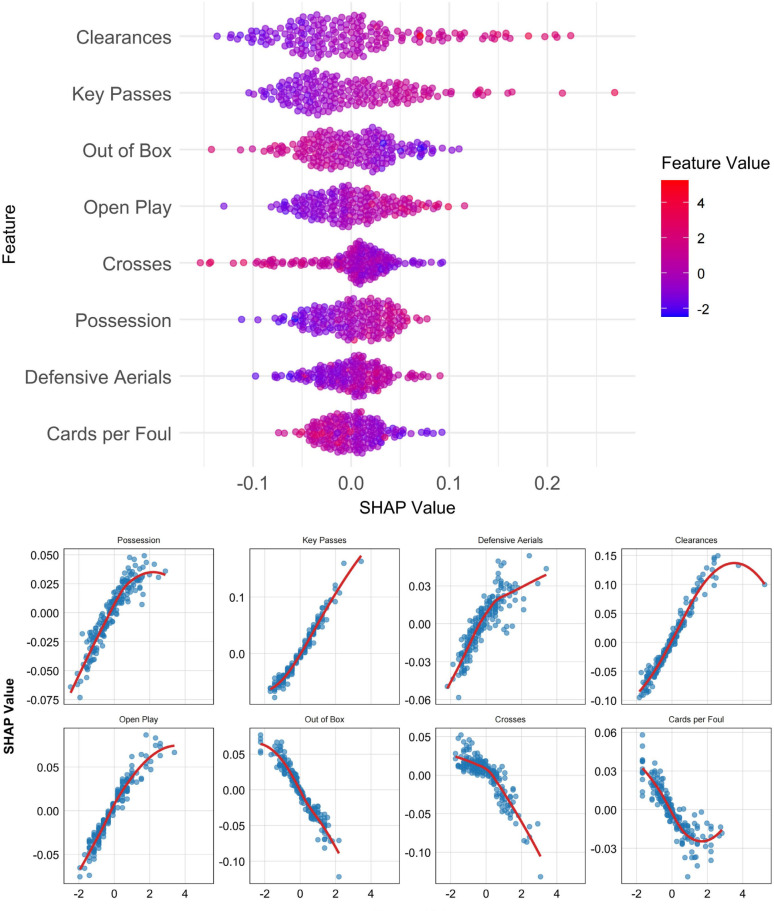
SHAP analysis of counterattacking style: feature importance and dependence relationships.

Figures 4 and 5 present SHAP value plots that illustrate the importance of individual features in predicting match outcomes for the two playing styles. Figure 4 displays SHAP-derived feature importance for the possession-based style using the SVM model, whereas Figure 5 presents the corresponding results for the counterattacking style using the KNN model. Features are ranked in descending order of influence, with the most important variables positioned at the top.

For the possession-based style (Figure 4), the top three predictive features were Key Passes, Crosses, and Possession. Features positively associated with winning outcomes included Crosses, Out of Box shots, Cards per Foul, and Offensive Aerials. Conversely, Key Passes, Possession, Aerials Won, Clearances, Accurate Passes, Tackle Success, Dribbles Won, and Defensive Aerials were negatively associated with match success.

For the counterattacking style (Figure 5), the three most important features were Clearances, Key Passes, and Out of Box shots. Indicators positively correlated with winning outcomes included Clearances, Key Passes, Open Play shots, Possession, and Defensive Aerials, whereas Out of Box shots, Crosses, and Cards per Foul were negatively associated with successful results.

## DISCUSSION

This study developed a model to classify UEFA Champions League teams into possession-based and counterattacking styles using an autoencoder for nonlinear dimensionality reduction and K-means clustering. Key features were selected through LASSO, Boruta, and XGBoost, and classification models (SVM, KNN, and LightGBM) were evaluated based on AUC and feature importance, with SHAP applied for interpretability. Compared with existing frameworks, this integrated pipeline combines nonlinear dimensionality reduction and complementary feature selection methods, improving both predictive performance and interpretability [34]. Although no direct performance comparison with prior studies was conducted, the results demonstrate the potential of this approach, which will require further validation in future work.

The classification revealed clear distinctions in performance indicators between possession-based and counterattacking teams. Possession-based teams exhibited higher values in passing-related metrics and placed less emphasis on defensive actions, consistent with their focus on ball retention and structured, accurate, and repeatable passing sequences [7]. Pass frequency and accuracy remain critical indicators for such teams [7], and players typically demonstrate higher technical proficiency than those in counterattacking teams [9]. Counterattacking teams, by contrast, prioritize rapid exploitation of opponents’ defensive gaps, aiming to deliver the ball quickly to well-positioned teammates immediately after regaining possession [35]. This results in shorter possession phases, fewer passes, and a greater reliance on long forward passes [9].

Analysis of key performance indicators (KPIs) identified Possession, Key Passes, Defensive Aerials, Clearances, Out of Box shots, Crosses, and Cards per Foul as common predictors for both playing styles. However, their impact on match outcomes differed significantly across the two styles, underscoring the fundamental tactical contrasts. For possession-based teams, Out of Box shots, Crosses, and Cards per Foul showed positive associations with winning probability, whereas most other indicators had negative associations. Key Passes, Crosses, Possession, Out of Box shots, and Cards per Foul emerged as the five most important predictors of match outcomes. Contrary to conventional assumptions, higher Key Passes and Possession, which are commonly regarded as hallmarks of successful possession-based play, were linked to an increased likelihood of losing. Although high possession theoretically reflects control over the game [36], excessive possession combined with frequent key passes may indicate inefficiency in breaking down compact defenses and low conversion rates from shooting opportunities. The nature and trajectory of key passes are therefore crucial, as previous studies have shown that pass type strongly influences shot success [37]. Research also suggests that teams effectively circulating the ball horizontally in the attacking third are more likely to create high-quality scoring chances [7, 38, 39]. This aligns with the present findings, where a higher number of crosses correlated with increased winning probability for possession-based teams. Excessive central penetration attempts and prolonged possession phases may thus reduce scoring efficiency, while wide-play strategies, such as frequent crossing, appear more effective in generating goal-scoring opportunities.

Additionally, Out of Box and Cards per Foul were identified as common key indicators for both playing styles, exerting a positive influence on match outcomes for possession-based teams. Studies have suggested that for possession-oriented teams, an increased number of passes tends to reduce shooting frequency [40]. Excessive passing may therefore limit shot opportunities and lower the probability of winning. Rather than relying solely on intricate passing sequences aimed at penetrating the penalty area, possessionbased teams should exploit long-range shooting opportunities when available. Diversifying offensive strategies with attempts from outside the box can enhance attacking efficiency and increase scoring potential. Cards per Foul also serves as an important measure of tactical discipline. Although a higher number of cards might initially seem detrimental, this metric should be interpreted within the broader match context, as it can reflect factors such as match intensity, officiating style, and deliberate tactical choices. For possession-based teams, committing strategic fouls is often a deliberate approach to disrupt opponents’ counterattacks and prevent dangerous transitions [41].

By contrast, the key indicators for counterattacking teams show an inverse pattern. Notably, Possession and Key Passes are positively associated with match outcomes, while Crosses and Out of Box shots exhibit a negative relationship. As discussed earlier, counterattacking teams focus on exploiting defensive gaps through direct, high-impact transitions [9]. Due to limited possession time, these teams must maximize each offensive opportunity by prioritizing efficiency over volume. In this context, indiscriminate crossing or longrange shooting may reduce scoring probability and disrupt the pace and direction of transition-based play. Among the eight key indicators identified for counterattacking teams, Clearances stands out as a critical defensive metric. In contrast to possession-based teams that emphasize control and structured buildup, counterattacking teams depend on swift transitions following defensive actions. Clearances help remove the ball from high-risk areas, disrupting the opposition’s attack and creating opportunities for counterplay [42]. This proactive defensive approach limits exposure to dangerous situations and protects against conceding goals. Cards per Foul was negatively associated with match outcomes in counterattacking teams. Since their defensive positioning is often deep in their own half, committing fouls in these areas heightens the risk of conceding from set pieces or penalties. To maintain defensive integrity and minimize risk, such teams must avoid fouls in critical zones near the goal.

These findings indicate that counterattacking teams achieve favorable outcomes not merely through deep defensive positioning but by transforming defensive actions into purposeful transitions and exploiting spatial imbalances during ball recovery. Possession-based teams, in contrast, dominate ball circulation but often struggle to convert sustained control into high-quality scoring opportunities, particularly against compact, well-organized defensive structures. The interaction patterns further suggest that wide-channel attacks are most effective when combined with timely central penetration, especially within the penalty area.

The tactical distinctions observed in this study corroborate and extend previous research on possession-based and counterattacking styles in elite competitions. Studies on La Liga teams have documented a trend toward longer passing sequences with high accuracy, accompanied by slower progression and reduced penetration into the final third [43]. This is consistent with our observation that possession-based teams increasingly depend on crosses and long-range shots to break down compact defenses. Such reliance reflects a broader tactical dilemma: converting sustained possession into effective offensive output. A similar issue was evident in the 2018 FIFA World Cup, where possession-based teams, despite outperforming in goalrelated, attacking, and passing metrics, often failed to translate their dominance into decisive scoring opportunities [44].

Research on top women’s national teams during major international tournaments has also emphasized the importance of balanced possession strategies—integrating cautious buildup with timely forward progression—as a characteristic of successful teams [45]. This aligns with our finding that tactical flexibility, rather than strict adherence to a single playing style, correlates more strongly with positive outcomes. Collectively, evidence from men’s domestic leagues, women’s international tournaments, and elite continental club competitions supports the conclusion that success depends not on possession quantity alone but on its efficiency, decisiveness, and contextual adaptation.

### Limitations and Future Directions

This study has several limitations. It relied on static, aggregated match data that do not capture dynamic in-game events, such as tactical shifts or momentum changes. Contextual factors including opponent strength, match location, and player availability were not incorporated, despite their likely influence on tactical choices and outcomes. The data structure did not account for nesting effects (e.g., repeated team observations across seasons), which may violate independence assumptions. Moreover, while SHAP improved model interpretability, the binary classification of outcomes (win vs. non-win) limited the tactical relevance of the findings, as coaches often consider more nuanced outcomes, such as draws, goal difference, and phase-specific performance (e.g., pressing, transitions).

Future research should integrate time-series or event-level data to capture the dynamic nature of football matches and improve sensitivity to tactical adjustments. Contextual variables such as opponent strength, match location, and player availability should be included to enhance predictive robustness and practical applicability. Employing hierarchical or mixed-effects models would better address the nested data structure, improving the rigor of inference. Adopting more granular outcome measures and linking model interpretations to in-game actions and tactical phases will further enhance relevance for practitioners. Finally, the proposed analytical framework demonstrates strong extensibility and can be applied to women’s football, enabling comparative analyses between men’s and women’s competitions to better understand gender-specific tactical characteristics.

## CONCLUSIONS

This study developed a data-driven framework to classify UEFA Champions League teams into possession-based and counterattacking styles and to predict match outcomes based on performance indicators. Distinct performance profiles were observed for each tactical orientation. Possession-based teams achieved better results when combining ball control with effective long-range shooting, whereas excessive reliance on possession and key passes was associated with reduced efficiency. Counterattacking teams performed best when integrating efficient passing with defensive robustness, with Clearances emerging as a key differentiator.

The findings offer practical implications for coaching and match analysis. Selected indicators such as long-range shots, key passes, and clearances can be integrated into opposition scouting and postmatch reviews. For possession-based teams, training should emphasize purposeful ball circulation and high-quality shot creation rather than passing volume alone. Counterattacking teams should prioritize compact defending, efficient transitions, and minimizing fouls in high-risk zones. Indicators such as Clearances hold predictive value across both styles and can inform defensive training emphases. These insights can support scenario-based tactical planning and real-time adjustments in response to opponents’ behaviors, provided that model outputs are interpreted within the limits of correlational inference.
